# Do individual liquor permit systems help Indigenous communities to manage alcohol?

**DOI:** 10.1111/dar.12994

**Published:** 2019-10-27

**Authors:** Peter d'Abbs, Ian Crundall

**Affiliations:** ^1^ Substance Misuse Studies Menzies School of Health Research Darwin Australia; ^2^ Strategic Dimensions Brisbane Australia

**Keywords:** liquor permits, Indigenous communities, Indigenous population, alcohol drinking, community development

## Abstract

**Introduction and Aims:**

Liquor permits were once used throughout Scandinavia and North America for managing alcohol, but largely disappeared in the late 20th century. Today, they are used in some Indigenous communities in Nunavut, Canada and the Northern Territory, Australia. This paper examines the extent to which liquor permits: (i) contribute to reducing alcohol‐related harms in Indigenous communities; and (ii) offer a viable mechanism for managing alcohol in Indigenous communities.

**Design and Methods:**

The study draws on published and unpublished international literature on liquor permit systems in Indigenous communities, and on field visits to northern territory (NT) communities.

**Results:**

Apart from one anecdotal report, the study found no evidence that liquor permit systems in Nunavut communities have reduced alcohol‐related problems. In the NT, they have reduced alcohol‐related harms in some communities. However, management of liquor permit systems generates significant administrative demands in communities.

**Discussion and Conclusions:**

Effectiveness of liquor permit systems is a product of five factors: permits themselves; agencies and procedures for issuing and managing permits; agencies and procedures for supplying liquor; enforcement of permit conditions, and the presence of other agencies—legal and illegal—affecting supply and consumption of liquor. Liquor permits continue to be valued by some Indigenous communities for managing alcohol. This study suggests that they can do so provided: (i) agencies administering permits have adequate support; (ii) controls over non‐legal purchasing and consumption of liquor are effective, and (iii) the permit system is viewed in the community as legitimate, equitable and transparent.

## Introduction

Liquor permit systems represent a distinctive type of measure for managing alcohol. Unlike more widely used measures such as minimum drinking age, licensing conditions and taxes, they target specific individuals. They are not widely used today, although in the early 20th century they were prominent in alcohol control policies in Canada, some US states and Scandinavia. Room 1 has reviewed literature generated by these systems and concludes that, despite permit systems by their very nature generating considerable amounts of data, very little research was carried out to assess their impact, and the few studies that were conducted were inconclusive. From the 1950s, liquor permit systems were progressively abandoned in most of Scandinavia and North America, having come to be seen as intrusive, discriminatory and ineffective. Although they are still used in some states of India 2, liquor permits are rarely discussed today in policy literature. For example, a 2009 World Health Organization report on strategies to reduce the harmful use of alcohol makes no mention of them 3. Babor *et al*.'s comprehensive review of policy approaches in *Alcohol: No Ordinary Commodity* 4 is similarly silent on individualised permit systems.

One domain in which liquor permits continue to be deployed, however, is that of remote Indigenous communities—especially in the Northern Territory (NT) of Australia and the territory of Nunavut in northern Canada. In both jurisdictions, liquor permit systems have been in place since the 1980s as legally recognised mechanisms for local community control over alcohol use. In this paper, we review the evidence relating to the operations and impact of liquor permit systems in both jurisdictions. The paper addresses two questions:

1. To what extent do individual liquor permit systems help to reduce alcohol‐related harms in Indigenous communities?
2. To what extent do liquor permit systems offer a viable mechanism for community‐based management of alcohol in Indigenous communities?

## Methods

The analysis reported in this paper originated from a consultancy funded by the then NT Department of Business to examine operations of liquor permit systems in the NT and develop guidelines for future use. (Reports produced under the project are available 5, 6.) One component of the project was a narrative review of international literature relating to liquor permit systems based on a search of the following databases: AIATSIS Indigenous studies bibliography; Anthropological index online; CINCH Australian criminology database; DRUG database; Google Scholar; Medline; PsychINFO; Sociological abstracts; and Web of Science. Search terms used were ‘liquor permit*’, ‘alcohol permit*’, ‘grog permit*’, ‘permit system*’. Two other components of the project were, firstly, an examination of files held by the NT Liquor Commission and, secondly, field visits that we conducted in each of the 22 NT Indigenous communities with liquor permit schemes in 2015 and 2016. We interviewed individuals and groups involved in the operation of liquor permit schemes including, where they existed, local permit committees. The objectives in gathering data from these sources were to describe the origins, evolution and impact of liquor permit systems in communities, and to document operational issues and steps taken to address them.

Both the file review and fieldwork components of the study were restricted to the NT. Data relating to Nunavut discussed in this paper is drawn from the literature review only. We recognise that this limits our knowledge of liquor permit systems in Nunavut compared with the NT, but believe that a comparative analysis of the two settings, drawing on the data available here, is both feasible and useful for understanding the place of liquor permits in community‐based management of alcohol.

The project received Ethics Approval (Ref 2015‐2528) from the Human Research Ethics Committee of the NT Department of Health and Menzies School of Health Research.

## Results

In this section we describe, firstly, the operation and impact of liquor permit systems in Nunavut, followed by a description of liquor permits in the NT. [Throughout the paper, we use the term ‘Indigenous’ to refer to indigenous peoples of the regions under review, and follow the current Australian convention of capitalising the term. We are aware that several other terms are also commonly used, such as Aboriginal, First Nations and (in Canada) Metis, as well as terms by which particular people refer to themselves. Our usage is not intended to signify a preference for one term over others or to gloss distinctions conveyed by these terms, but to lend consistency and clarity to our analysis.]

### *Liquor permits in Nunavut, Canada*

The territory of Nunavut comprises one‐fifth of Canada's landmass and is occupied by just 38 456 people, of whom 84% are Inuit—the Indigenous occupants of the region 7. A Nunavut Government report in 2016 observed that, while Inuit people were less likely than other Canadians to drink alcohol at all, they experienced high levels of alcohol‐related harms, which were exacerbated by factors such as overcrowding, low incomes, limited education and intergenerational trauma 8. A study of the costs and harms of substance use in Canadian provinces and territories reported that the per capita cost of substance use in Nunavut in 2014 was $2652—more than double the national level of $1081 and higher than any other jurisdiction. About 45% of the Nunavut cost was attributed to alcohol 9.

The supply of alcohol in Nunavut is shaped by constraints of climate and remoteness. Transport of liquor to Nunavut by sea is possible only for a few months of the year, and otherwise must be by air. Within Nunavut it is delivered by air. Liquor for retail sale is purchased by the government and distributed through the Nunavut Liquor Commission (NLC), which maintains two warehouses in the territory. Under the Nunavut *Liquor Act*, the 25 communities scattered throughout the territory may choose—by local plebiscite—one of four systems for controlling alcohol:

1. An unrestricted system in which the community is subject only to the general liquor laws of Nunavut;
2. A restricted quantities system in which the quantity of liquor that a person may purchase is limited;
3. A committee system, in which a locally elected Alcohol Education Committee (AEC) decides who may import, possess, consume and/or purchase liquor in the community, and the conditions under which they may do so. The AEC is also expected to provide education and counselling services;
4. A prohibition system 10.

As of July 2018, 13 communities had AECs, six had prohibition and the remaining six were unrestricted 11.

Purchases of alcohol by individuals in Nunavut are subject to two kinds of permits: Liquor Permits and Import Permits. Liquor Permits allow approved individuals in restricted communities to purchase alcohol from one of the two warehouses run by the NLC, subject to any limitations that the community's AEC may impose. Permit holders must complete a Purchase Order for each purchase, which must be endorsed by the AEC before being forwarded to the warehouse, from where the order will be despatched by air to the community 12. Individuals in restricted or unrestricted communities may also import small amounts of liquor from outside Nunavut. Anyone wishing to import larger amounts, however, requires an Import Permit, for which the NLC charges a fee. Residents of communities where alcohol is prohibited are not eligible for either kind of permit 11.

Evidence about the effectiveness of AEC‐based systems is limited and inconclusive. An anecdotal report from one community—Kugluktuk—quoted police as attributing a decline of 30% in reported incidents in the 12 months following the formation of an AEC in 2007 to the associated liquor restrictions 13. In another community, however, the introduction of a permit system in 2012 was said 12 months later to have made little difference to ‘bootlegging’, or the illegal importation of liquor 14. Wood examined records of homicide, serious assaults and sexual assaults in 23 Nunavut communities between 1986 and 2006 15. He found that rates in ‘dry’ (i.e. prohibition) communities were significantly lower than in either unrestricted or restricted communities, though even here they were above national rates. However, he found little difference between restricted and unrestricted communities, with the former recording 64 violent crimes per 1000 persons, the latter, 67 violent crimes per 1000 persons.

A government task force appointed in 2010 to review operations of the *Liquor Act* was repeatedly told in consultations that current control systems were not working and that AEC members lacked the resources to perform either an educational or control function 16. One committee member stated: ‘No one has provided us with the proper education on alcohol so how can we make good decisions and be expected to educate others?’. The permit system for controlling importation of liquor into communities from outside Nunavut was also said to be exploited by bootleggers, who on occasions utilised flaws in the surveillance system to make multiple purchases using a single Purchase Order. Despite these criticisms, the task force found that people in communities were ‘united in believing that AECs had an important role in supporting community well‐being’ [16, p. 29].

The task force proposed a re‐orientation of Nunavut alcohol policy with the aim of reducing consumption of illegally obtained spirits and liberalising access to wine and beer. It recommended abolishing Import Permits and establishing a government monopoly over the importation, sale and distribution of alcohol throughout Nunavut 12. While it recommended retaining the system under which AECs in restricted communities issued Liquor Permits, it called for responsibility for approving orders for individual purchases to be transferred to the Liquor Commission. It also recommended that AECs be established in all communities—including those where alcohol was prohibited—with adequate resources and support from a central secretariat. Finally, the task force proposed that, subject to the approval of communities concerned, the government open or licence beer and wine stores in communities.

In response to the task force, the government introduced an action plan in October 2016 8. While the government undertook to improve permit systems and increase resources for AECs, it did not establish a government monopoly. It did, however, liberalise access to beer and wine—at least in the community of Iqaluit. In September 2017, following a plebiscite in which 77% of participants voted in favour of the proposal, the Iqaluit Beer and Wine Store opened as a three‐year pilot project. The NLC claims that in its first year of operation the store brought about a ‘drastic’ fall in sales of spirits, which served to offset an increase in beer and wine sales 17. The Commission's own figures, however, indicate that, while sales of spirits indeed declined by 12.8%, those of wine and beer grew by 350% and 73%, respectively. As a result, the amount of liquor sold in Iqaluit increased from 511 958 L in 2016–2017 to 934 492 L in 2017–2018. When beverage categories are converted to pure alcohol, this represents an increase of 73.8%. These figures do not take into account what some observers have identified as high levels of unrecorded alcohol sales in Nunavut in previous years 18, and in any case it is too soon to determine what impact, if any, they might have on harm indicators. Since Iqaluit is not a restricted community, the presence of a new beer and wine outlet does not have implications for liquor permit systems in other Nunavut communities.

### *Liquor permits in Northern Territory of Australia communities*

The introduction of liquor permits in Indigenous communities in the NT in 1980 grew out of a shift in government policies affecting Indigenous minorities that occurred in Australia and elsewhere through the 1960s and 1970s 19, 20, 21. As part of the shift, Australian jurisdictions dismantled long‐standing laws forbidding Indigenous Australians from consuming alcohol.

The NT is a sparsely‐settled, largely tropical region occupying around one‐sixth of the Australian landmass, with an estimated resident population in 2016 of 228 833 persons, of whom 25.5% were Indigenous 22. Many Indigenous people live in remote communities 23. Prior to 1980, control over liquor in these communities was vested in non‐Indigenous superintendents. A new *Liquor Act* introduced in 1980 allowed communities themselves to stipulate the conditions under which alcohol was to be made available—subject to the general liquor laws of the NT—or whether it should be made available at all 24. One option available to communities that wished to restrict but not ban alcohol was to grant permits to approved individuals, specifying the amount and types of alcohol they could bring into the community, and where they could drink it. Liquor permits were to be issued by the NT Liquor Commission, a statutory body set up to administer the Act, on advice from the local council and the police. Although the NT Liquor Act has subsequently been amended several times, the provisions governing liquor permits remain substantially unaltered. (In the *Liquor Act 2019* they occupy sections 198‐204 25.)

Today, most Indigenous communities in the NT—96 communities—are located within what are now known as General Restricted Areas, but the majority of these (74 communities) make no provision for liquor permits. In the 22 remaining General Restricted Areas, liquor permit schemes have evolved on an *ad hoc* basis. Previous reviews identified a number of operational problems 26, 27, including heavy paperwork demands, confusion over the relative responsibilities of communities, police and the licensing authority, inadequate monitoring by the licensing authority and, in some instances, resentment among community members regarding what they perceived to be race‐based discrimination in the granting of permits 26, 27, 28, 29.

Our review found that liquor permit systems could be categorised as one of two types. In the first, liquor permits serve as a mechanism to enable employees in communities—mostly non‐Indigenous employees—to import and consume liquor in their own homes in what would otherwise be dry or heavily restricted communities. In principle any adult resident of a community is entitled to apply for a permit. In practice, while non‐Indigenous residents obtain liquor permits more or less routinely, for Indigenous residents the pathway is generally neither clear nor smooth. In these communities, liquor permits are a peripheral rather than a core part of the local provisions for managing alcohol. Nonetheless, they tend to generate various operational issues and problems, which we discuss below. Because their prime purpose is to exempt approved individuals from restrictions that apply to everyone else in a community, we labelled schemes of this type *exemption* schemes. Of the 22 communities operating liquor permit schemes, 12 fell into this category.

The second type of liquor permit scheme, far from being a peripheral mechanism, constitutes the main means for managing local alcohol use. Liquor permits are given to local Indigenous and other residents to encourage moderate consumption and thereby minimise alcohol‐related harm. Recommendations regarding permits are made by a local liquor permit committee (LPC), vetted by local police, and enacted by the Licensing Commission. The LPC can also recommend revocation or amendment of permits. We labelled schemes of this type *permit‐based alcohol management systems*. We identified six systems of this type, involving between them 10 communities. Three schemes are located on the Tiwi Islands north of Darwin, the capital city of the NT, and another is at Maningrida in Arnhem Land. The remaining two schemes are based on Groote Eylandt and the Gove Peninsula, respectively. In this paper, we confine our discussion to these two schemes, in part because they have both been shown in independent evaluations to have reduced alcohol‐related harms, and in part because their innovative use of modern communications technology makes them interesting case studies for possible application elsewhere.

The Groote Eylandt and Bickerton Island Alcohol Management System (GEBIAMS) and the Gove Peninsula Alcohol Management Plan both use liquor permits to regulate *purchases* of takeaway (packaged) liquor, rather than alcohol importation or consumption. Both systems incorporate networked, electronic point‐of‐sale ID systems linked to a central server in Darwin. The GEBIAMS commenced in July 2005 30. Groote Eylandt (Dutch for ‘big island’) lies in the Gulf of Carpentaria, approximately 600 km by air east of Darwin. It contains three major settlements—the Indigenous communities of Angurugu and Umbakumba, and the mining town of Alyangula—as well as several smaller settlements. The total population is just under 2500 persons, of whom 65% are Indigenous 31. At the time the system commenced, Groote Eylandt had two takeaway liquor outlets, one of which has since closed. It also had a history of alcohol‐related problems dating back to the start of manganese mining on the island in the 1960s 32. Under GEBIAMS, it is illegal to buy takeaway liquor without a permit. On‐premise sales are also available on the island at two outlets, but only to members of the Alyangula Golf Club in one case and, in the other, to permit holders. Applications for a permit are considered by a local LPC, which includes representatives from Anindilyakwa Land Council, the three main communities, liquor outlets, the mining company, police and health services, and a consumer representative 32. The committee makes recommendations to the Liquor Commission.

A striking feature of GEBIAMS is the extensive community engagement that lay behind it 30. An independent evaluation of its first 12 months of operation found strong evidence of beneficial outcomes 32. Compared to the preceding year, recorded assaults and aggravated assaults fell by 73% and 67%, respectively, and the number of persons placed in ‘protective custody’ for being publicly intoxicated fell from 90 to 11. The evaluators also found that the permit system was widely supported among Indigenous and non‐Indigenous residents alike. However, the evaluators also heard reports of high levels of cannabis use, which sometimes generated violence, especially when individuals ran out of supplies.

The Gove Peninsula Alcohol Management Plan commenced in 2008 and covers three major localities—the mining township of Nhulunbuy and the Indigenous communities of Yirrkala and Gunyangara—each of which is served by its own LPC. As with Groote Eylandt, takeaway purchases of liquor are prohibited without a liquor permit, but on‐premise sales are not restricted. A 2011 independent evaluation of the Gove Peninsula Plan found evidence of a decline in liquor sales, assaults, and alcohol‐related emergency department presentations 33.

### *NT liquor permit schemes in practice*

Both types of liquor permit system identified in the NT generated distinctive operational issues. In the case of exemption schemes, most of these derived from a decline over time of community input into decisions about liquor permits. None of the communities with exemption schemes had a functioning LPC. Despite the fact that, under legislation, all applications were supposed to be considered by the community, effective power to recommend permits had fallen by default to local police officers, who operated without administrative support or operational guidelines. In several communities, we were told that non‐Indigenous applicants routinely received liquor permits, while Indigenous applicants were treated harshly and sometimes capriciously. This in turn generated resentment. We found that the exercise of authority by police *per se* was not regarded as problematic; resentment rather arose from a perceived lack of transparency or consistency.

Turning to permit‐based alcohol management systems, while the limited evidence available suggests they can help to reduce alcohol‐related problems in a community, our review found they can also entail a heavy administrative burden. On both Groote Eylandt and the Gove Peninsula, this has been partly self‐imposed, as LPCs in both systems have created local ‘ladders’ of graduated permit levels. The Nhulunbuy permit ladder as shown in Table 1, for example, consists of six levels, beginning with a maximum takeaway purchase of six 375 mL cans of light beer (that is, beer containing <3% alcohol), and rising in a series of increments to an unrestricted permit.

**Table 1:** Graduated liquor permits, Nhulunbuy liquor permit committee^a^

Level	Permitted purchase (takeaway liquor): Choice of one dot pointed option at each level
1	6 × 375 mL cans light beer AND/OR one bottle wine.^b^
2	6 × 375 mL cans mid‐strength beer AND/OR one bottle wine. OR 12 × 375 mL cans light beer AND/OR one bottle wine.
3	6 × 375 mL cans full‐strength beer^c^ AND/OR one bottle wine. 12 × 375 mL cans mid‐strength beer AND/OR one bottle wine. 6 × 375 mL cans pre‐mixed drinks.
4	12 × 375 mL cans full‐strength beer AND/OR two bottles wine. 24 × 375 mL cans mid‐strength beer AND/OR two bottles wine. 12 × 375 mL cans pre‐mixed drinks AND/OR two bottles wine.
5	30 pack carton of full‐strength beer (375 mL cans) AND/OR two bottles wine. 30 pack carton of mid‐strength beer (375 mL cans) AND/OR two bottles wine. 24 × 375 mL cans pre‐mixed drinks (<5% alcohol) AND/OR two bottles wine.
6	Unrestricted.

a *Source*: https://nt.gov.au/law/alcohol/apply-for-an-individual-liquor-permit/apply-for-a-liquor-permit-in-east-arnhem.

b Use of the phrase ‘AND/OR’ is ambiguous, but is nonetheless present in LPC documents.

c Containing at least 4.5% alcohol by volume.

In Nhulunbuy (but not in some other communities), all new permits are unrestricted. However, should a permit holder be deemed to have breached the permit conditions, he or she is liable to have their permit revoked, with the period of revocation dependent on the seriousness of the offence. Any person who applies for re‐instatement must begin at the bottom rung of the ladder and work their way up by re‐applying, step by step, at intervals of not less than 1 month. All applications must be made in writing to the LPC, which considers them at its regular meetings.

Not all permit‐based alcohol management systems have graduated ladders. Even without these, however, recording, recommending and monitoring permits is administratively demanding for small community groups.

## Discussion

This examination of liquor permit schemes in two very different settings shows that, as instruments for managing alcohol use, permits cannot be assessed in isolation, but rather as components of what, for analytical purposes, constitute *systems* for managing the supply and consumption of alcohol. Key components of these systems are:

- permits themselves: that is, conditional authority to purchase, import and/or consume liquor, issued to approved individuals;
- agencies and procedures for issuing and managing permits;
- agencies and procedures for supplying liquor to permit holders;
- agencies and procedures for ensuring compliance with conditions attached to permits;
- other agencies and procedures (legal and illegal) affecting the supply and consumption of liquor in the locality covered by permits.

The effectiveness of a liquor permit system in any given community, we suggest, will be a product of the adequacy and suitability of each of these components. For example, evidence presented to the task force reviewing the Nunavut *Liquor Act* from 2010 indicated that AECs lacked the resources to oversee permit systems and this defect, combined with cumbersome procedures for purchasing liquor legally by using permits, created opportunities for ‘bootleggers’ to sell illicit liquor, usually spirits. Table 2 summarises key features of liquor permit systems in Nunavut and the NT in terms of the five components. (These components, we also suggest, are useful not only for analytical purposes, but also as a framework for communities considering setting up new permit systems.)

**Table 2:** Key components of liquor permit systems

Component	Nunavut, Canada	Northern Territory, Australia
Permits	Two types: (i) for purchase of liquor from government warehouses; (ii) for importing liquor from outside Nunavut.	Some permits regulate importation into community; some regulate purchasing; most apply to takeaway (packaged) liquor only.
Agencies and procedures for issuing and managing permits	Responsibility vested in community‐based Alcohol Education Committees, with Nunavut Liquor Commission also having an administrative role.	Permits issued, revoked, etc. by central Liquor Commission, acting on advice from local permit committees (where they exist) and local police. Some systems use electronic ID systems for checking customer's entitlement.
Agencies and procedures for supplying liquor	All liquor purchased within Nunavut sold by government; permits can be purchased to purchase liquor from outside the province.	Liquor supplied by private companies.
Agencies and procedures for ensuring compliance with permit conditions	Main responsibility lies with police.	Main responsibility lies with police.
Other agencies affecting supply and consumption of liquor	In the past, effectiveness of permit systems has been undermined by ‘bootlegging’ liquor.	In some communities with permits, effectiveness of permit systems in controlling alcohol use is undermined by availability of on‐premises liquor, which is not covered by permits.

As stated earlier, this paper seeks to answer two questions: (i) the extent to which liquor permit systems help to reduce alcohol‐related harms in Indigenous communities; and (ii) the extent to which they offer a viable means for community‐based management of alcohol in Indigenous communities. Regarding the first question, we have found no evidence that they reduce harms in Nunavut, other than an anecdotal report of a short‐term positive impact in one community. This may be due in part to the absence, so far as we are aware, of any rigorous evaluations of liquor permit systems in Nunavut. It may also be a result of defects in one or more of the key components identified in Table 2. In the NT, we have found no evidence that ‘exemption’ schemes have helped to reduce alcohol‐related harms. There is, however, evidence that permit‐based alcohol management systems can do so. Evaluations of the Groote Eylandt and Gove Peninsula systems, though both marked by the methodological limitations inherent in a simple pre‐test post‐test research design, found that alcohol‐related violence and other harms declined following introduction of the systems. Both systems, however, are located in distinct settings that may limit the degree to which they can be replicated elsewhere. Firstly, they are geographically isolated from major urban centres. Secondly, both settings are served by a small number of retail liquor outlets, making it relatively easy to monitor purchases.

Regarding the second question, the findings of the 2010 government inquiry in Nunavut might be seen as paradoxical: AECs were widely described as ineffectual in managing alcohol, but were also highly valued for their role in promoting community wellbeing, so much so that the task force conducting the inquiry recommended that they be set up in all Nunavut communities, regardless of their liquor status 12, 16. Taken together, these two observations can be read as testifying to a widespread desire in communities for an effective community voice in managing alcohol. In the NT, our research showed that in communities with exemption‐type liquor permit systems, community input into decision‐making had all but evaporated. This is likely to have been at least in part a product of the systems themselves: if the *de facto* function of an exemption scheme is to enable non‐Indigenous residents to drink liquor in otherwise dry communities, there is little incentive for Indigenous community members to make time available to participate in permit‐related processes.

In NT communities with permit‐based alcohol management plans, LPCs provide an institutional framework for community input into decision making. Exercising input, however, generates considerable administrative demands. This is consistent with observations regarding AECs in Nunavut communities 16. LPCs drawn from small communities, made up of a mix of *ex officio* members who have full‐time positions outside the committees, and local community members participating in a voluntary capacity, may struggle to meet the administrative demands these systems generate unless given adequate support from governmental or other agencies.

On Groote Eylandt and the Gove Peninsula, as we have seen, the administrative burden has been aggravated by the predilection of LPCs to set up complex systems for regulating individual access to takeaway liquor. Graduated permit ladders were not part of the alcohol management systems as originally approved by the Licensing Commission 30, 34. Their evolution appears to mark an extension of the role of LPCs from using liquor permits to control alcohol‐related harm at a community level to micro‐managing the drinking behaviour of targeted individuals. We know of no evidence that controls over takeaway liquor purchases lead to moderation of consumption, especially where, as in the Gove Peninsula, drinkers have unrestricted access to on premise liquor outlets.

Interestingly, the predilection for using individual liquor permits to control people's behaviour is not peculiar to LPCs in the NT. Room 1 describes permit systems in Sweden, Finland and Canada, where the administration of permit systems was accompanied by surveillance not only over individuals' liquor consumption, but also over their family, economic and social circumstances. In Canada, as Genosko and Thompson 35 show, these functions generated unwieldy bureaucracies that compiled extensive data on the drinking and other personal habits of citizens.

Our study has several limitations. Firstly, neither of us is Indigenous, nor do we live in Indigenous communities. Secondly, as we have shown, few liquor permit systems in Indigenous communities anywhere have been independently evaluated. Thirdly, while our examination of documentary sources regarding permit systems in the NT was complemented by fieldwork, we did not conduct fieldwork in Nunavut.

## Conclusions

Notwithstanding their apparent obsolescence in other settings, individual liquor permits continue to be valued in some Indigenous communities as a means of promoting community control over alcohol use. This study suggests that they can contribute to doing this—under certain conditions. The first is adequate administrative support: liquor permit systems create administrative demands both in communities and government agencies, and these demands need to be recognised and resourced. A second condition is the presence of effective controls over non‐legal purchasing and consumption. This in turn may be a product of remoteness from liquor outlets, surveillance of purchases, and/or efficient policing. A third condition is legitimacy in the eyes of the community—that is, a shared perception among community members that the system embodies the wishes of the community and that it is equitable and transparent.

## Conflict of Interest

The authors have no conflicts of interest.
